# Integrated proteomic and metabolomic analyses reveal the importance of aroma precursor accumulation and storage in methyl jasmonate-primed tea leaves

**DOI:** 10.1038/s41438-021-00528-9

**Published:** 2021-05-01

**Authors:** Jiang Shi, Jiatong Wang, Haipeng Lv, Qunhua Peng, Monika Schreiner, Susanne Baldermann, Zhi Lin

**Affiliations:** 1 Key Laboratory of Tea Biology and Resource Utilization of Ministry of Agriculture, Tea Research Institute, Chinese Academy of Agricultural Sciences, 9 South Meiling Road, Hangzhou, Zhejiang 310008 PR China; 2 Graduate School of Chinese Academy of Agricultural Sciences, 12 South Street of Zhongguancun, Beijing, 100081 PR China; 3 Leibniz Institute of Vegetable and Ornamental Crops, Theodor-Echtermeyer-Weg 1, 14979 Großbeeren, Germany; 4 University of Bayreuth, Food Metabolome, Faculty of Life Sciences: Food, Nutrition, Kulmbach, Germany

**Keywords:** Secondary metabolism, Metabolomics

## Abstract

In response to preharvest priming with exogenous methyl jasmonate (MeJA), tea plants adjust their physiological behavior at the molecular level. The whole-organism reconfiguration of aroma formation from the precursor to storage is poorly understood. In this study, we performed iTRAQ proteomic analysis and identified 337, 246, and 413 differentially expressed proteins in tea leaves primed with MeJA for 12 h, 24 h, and 48 h, respectively. Furthermore, a total of 266 nonvolatile and 100 volatile differential metabolites were identified by utilizing MS-based metabolomics. A novel approach that incorporated the integration of extended self-organizing map-based dimensionality was applied. The vivid time-scale changes tracing physiological responses in MeJA-primed tea leaves are marked in these maps. Jasmonates responded quickly to the activation of the jasmonic acid pathway in tea leaves, while hydroxyl and glycosyl jasmonates were biosynthesized simultaneously on a massive scale to compensate for the exhausted defense. The levels of α-linolenic acid, geranyl diphosphate, farnesyl diphosphate, geranylgeranyl diphosphate, and phenylalanine, which are crucial aroma precursors, were found to be significantly changed in MeJA-primed tea leaves. Green leaf volatiles, volatile terpenoids, and volatile phenylpropanoids/benzenoids were spontaneously biosynthesized from responding precursors and subsequently converted to their corresponding glycosidic forms, which can be stably stored in tea leaves. This study elucidated the physiological response of tea leaves primed with exogenous methyl jasmonate and revealed the molecular basis of source and sink changes on tea aroma biosynthesis and catabolism in response to exogenous stimuli. The results significantly enhance our comprehensive understanding of tea plant responses to exogenous treatment and will lead to the development of promising biotechnologies to improve fresh tea leaf quality.

## Introduction

Jasmonates (JAs; also known as jasmonic acid and its derivatives) are a large family of lipid-derived plant metabolites that mediate responses to stress and regulate development. The methylated derivative methyl jasmonate (MeJA) is a vital cellular regulator that modulates diverse developmental processes and defense responses against biotic and abiotic stresses^1–3^. Considered an efficient elicitor, MeJA has been widely applied exogenously to improve the quality of horticultural crops, especially fruits. Examples of fruits in which the flavor has been apparently enhanced include peach^4^, strawberry^5^, and loquat^6^. Similar improvements after priming with MeJA for a short period of time have also been observed in vegetables and other crops, such as sweet basil^7^, tomato^8^, tobacco^9^, and rice^10,11^. Exogenous application of MeJA to plants plays a significant role in activating the JA pathway, leading to a series of multidimensional responses extending from genes to proteins and eventually to secondary metabolites.

Tea (*Camellia sinensis* (L.) O. Kuntze) is an important economic crop in numerous countries around the world and has become a popular beverage worldwide, exhibiting an elegant flavor and health-promoting effects^12^. The quality of tea, which contributes to its commercial value, is mainly defined by its aroma and taste^13^. Well-characterized nonvolatile secondary metabolites (mainly polyphenols, flavonoids, theanine, alkaloids) are responsible for its taste^14^, and volatile secondary metabolites (volatile terpenoids, phenylpropanoids/benzenoids, and fatty acid derivatives) are fundamental to its aroma^13^. These secondary metabolites are biosynthesized in various complex pathways, among which, α-linolenic acid degradation leads to the formation of green leaf volatiles and MeJA. Monoterpenes (C10), sesquiterpenes (C15), and diterpenes (C20) are formed by specific enzymes within the terpenoid biosynthesis pathways, and volatile phenylpropanoids/benzenoids (VPBs), such as benzyl alcohol, benzaldehyde, 2-phenylethyl alcohol, and phenylacetaldehyde, are biosynthesized from the precursor shikimic acid and have already been widely investigated^15^. The importance of the stress response for the biosynthesis of secondary metabolites and flavor formation is well characterized^16^.

As a perennial evergreen woody crop, tea plants are constantly exposed to biotic and abiotic stresses, including herbivorous insects^17^, environmental changes^18^, and even exogenous elicitor inducement^19^. These factors are known to induce high variations in the levels and composition of the secondary metabolism that determine the final quality of tea. However, the exact mechanism by which the abovementioned secondary metabolites in tea respond to (a)biotic stress in tea remains unknown. Considering the complexity of secondary biosynthesis in tea plants, very simple methodologies cannot satisfactorily explain these variations. In recent years, “omics” approaches have allowed high-throughput analyses of the responses induced by environmental stress at the molecular level, thereby confirming data previously obtained with targeted analyses and extending the scope of investigation^20^. With the continuous and dynamic development of different strategies to study tea physiology, three predominant approaches have emerged. Transcriptomic or proteomic approaches revealed the differentially expressed genes or proteins in tea plants during cold acclimation and in response to drought stress^21,22^. By a metabolomic approach, changes in valuable metabolites, including volatiles and nonvolatiles, could be uncovered^23^. However, each individual “omics” approach elucidates only a part of the physiological responses. The only solution to comprehensively understand the complexity of the dynamic changes in secondary metabolism is the application of an integrated “multiomics” approach^24,25^.

According to our previous results, the application of MeJA to fresh tea leaves significantly enhanced the aroma quality of prepared teas^26^. Previously, we conducted combined transcriptomic and volatile metabolite analyses and partially elucidated physiological changes at the molecular level^27^. Although general response pathways were successfully elucidated in our studies, the out-of-sync differential expression of genes and volatile metabolites in MeJA-primed tea and the accumulation of aromas were not elucidated. It is meaningful and crucial to explore how plant proteins respond to MeJA priming, a known modulatory process before metabolite biosynthesis, in tea leaves. Hence, we adopted a promising “omics” strategy that combines iTRAQ-based proteomics and MS-based metabolomics to study the effects of MeJA priming on tea leaves, especially to elucidate the important floral aroma biosynthesis pathways. Using different time spans, we facilitated the characterization of dynamic life processes occurring in tea leaves at the molecular level by investigating protein-to-metabolite dynamics. This research lays a solid foundation for future investigation of the physiological and ecological functions of *C. sinensis* floral components on the basis of precursor formation and storage under exogenous treatment, with the promise of higher-quality fresh tea leaves. Moreover, the accumulation of these precursors could be meaningful for more sustainable tea production, as they could also serve as precursors of natural repellants^3,15–17^.

## Results

### Quantitative proteomic profiling of MeJA-primed tea leaves by iTRAQ labeling

We subjected MeJA-primed tea leaves to proteomic analysis to elucidate changes, as described in detail in Fig. S1. A total of 66,596 spectra were obtained, of which 48,536 were unique. We identified 21,546 peptides and matched 17,747 of them with known sequences from our iTRAQ proteomic results. Finally, 8106 proteins were identified, as presented in Fig. S2. The average molecular mass of the identified proteins was between 10 kDa and 70 kDa, and the protein sequence coverage was higher than 5% for 76.75% of the proteins. The identified proteins were classified according to gene ontology (GO) analysis into the following three main categories: biological processes, cellular components, and molecular functions (Fig. S3A). Furthermore, cluster of orthologous groups of proteins (COG) analysis showed that proteins involved in “posttranslational modification, protein turnover, chaperones” were mostly affected by MeJA priming (Fig. S3B). Additionally, protein sequences encoding enzymes of major significance for flavor formation, such as those involved in “amino acid transport and metabolism”, “lipid transport and metabolism”, and “secondary metabolite biosynthesis, transport, and catabolism”, were identified and further investigated.

### Differentially expressed proteins (DEPs) in MeJA-primed tea leaves

**Fig. 1:**
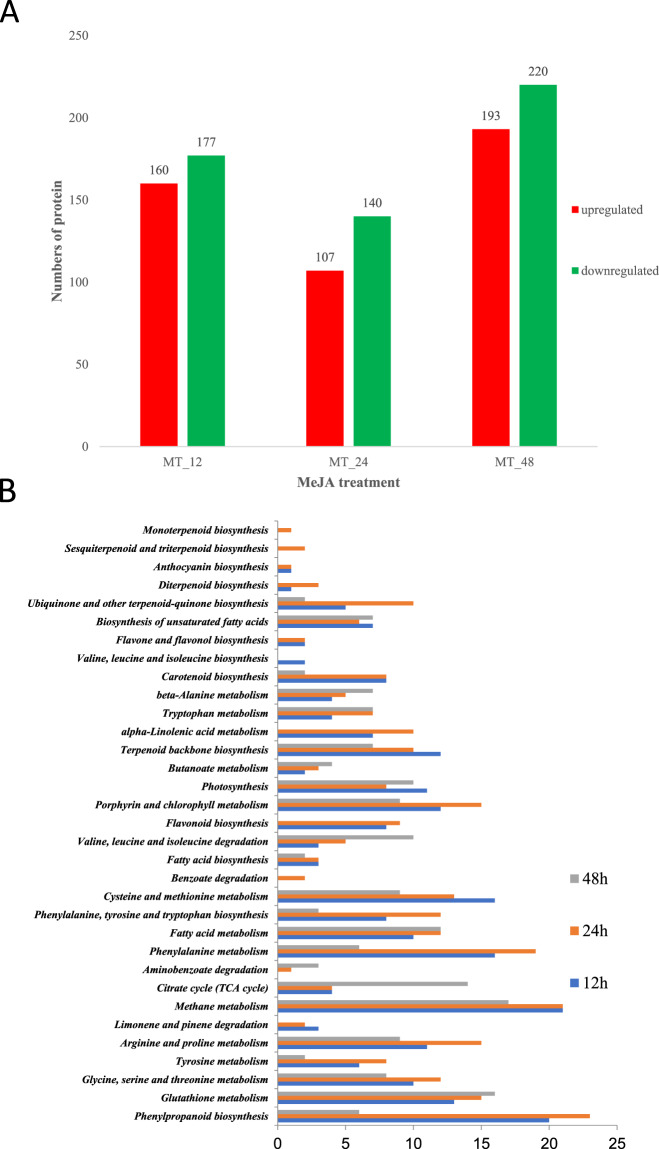
Differentially expressed proteins (DEPs) identified by iTRAQ-based proteomics in MeJA-primed tea leaves. **A** Differentially up- and downregulated proteins in MeJA-treated fresh tea leaves after 0, 12, 24, and 48 h. **B** Enrichment of the most significant pathways based on the evaluation of the iTRAQ results. Thirty-three pathways involved in secondary metabolism in *C. sinensis* were classified according to their DEPs: 12 h, samples after 12 h of MeJA priming; 24 h, samples after 24 h of MeJA priming; 48 h, samples after 48 h of MeJA priming

First, proteins with a fold change <0.85 or >1.25 were generally classified as DEPs (*p* value <0.05). In total, 996 DEPs were identified (Fig. 1A; detailed protein information is shown in Table S1). We identified 160, 106, and 193 upregulated proteins in samples primed with MeJA for 12 h, 24 h, and 48 h, respectively, and identified 177 (12 h), 140 (24 h), and 220 (48 h) downregulated proteins in these samples, respectively.

To gain an in-depth understanding of the changes in volatile-related metabolic pathways in response to MeJA priming, comprehensive investigation of the DEGs involved was performed. Thirty-three secondary metabolic pathways were identified (Fig. 1B), in addition to the α-linolenic acid degradation pathway (23 DEPs), terpene backbone biosynthesis (42 DEPs), and phenylalanine-related metabolism (43 DEPs).

#### Crucial DEGs involved in the LOX pathway

Three different lipoxygenases (LOXs), namely, lipoxygenase 2 (LOX2S), lipoxygenase 3 (LOX3S), and lipoxygenase 9 (LOX9S), associated with the formation of green leaf volatiles (GLVs), were identified in MeJA-primed tea leaves. In particular, the levels of LOX2S and LOX3S exhibited significant increases in the MeJA-primed samples (1.3- to 2.1-fold, Table S1), corresponding to changes in this class of volatiles. Additionally, the expression levels of other enzymes directly or indirectly involved in flavor formation pathways increased, including those of phosphatase, allene oxide synthase (AOS), allene oxide cyclase (AOC), and 2-oxophytodienoate reductase (OPR). For the first time, we identified two potential jasmonic acid O-methyltransferase (JMT) enzymes in tea. Interestingly, the level of JMT1 increased (2.2-, 1.5-, and 1.5-fold after 12, 24, and 48 h, respectively), while JMT2 displayed the opposite trend (0.8-, 0.6-, and 0.7-fold after 12, 24, and 48 h, respectively).

#### Crucial DEGs involved in the volatile TP biosynthesis pathway

A key enzyme related to the terpenoid (TP) backbone biosynthesis pathway, 1-deoxy-D-xylulose-5-phosphate synthase (DXS), was enriched by MeJA treatment, showing 3.7 (12 h), 2.4 (24 h), and 1.8 (48 h) times higher expression levels. The levels of geranylgeranyl diphosphate synthase (GGPS) showed a significant decrease in primed samples, whereas farnesyl diphosphate synthase (FPS) and geranyl diphosphate synthase (GPS) were enriched in the 12 h samples. This was an indication of increased biosynthesis of precursors for the formation of volatile TPs. Furthermore, the level of nudix, which is considered the core enzyme in the initial hydroxylation of phosphate groups, changed significantly in the MeJA-primed samples. In addition, the expression of phosphatases possibly catalyzing a second hydroxylation of phosphate groups (GP, FP, GGP) apparently declined. MeJA priming also changed the levels of key enzymes in the nonmevalonate pathway. Specifically, in the carotenoid metabolism pathway, six key DEPs (zeta-carotene desaturase, ZDS; cytochrome P450-beta-ring hydroxylase, LUT5; carotene epsilon-monooxygenase, LUT1; carotenoid cleavage dioxygenase 1, CCD1; xanthoxin dehydrogenase, ABA2; and abscisic-aldehyde oxidase, AAO3) were tagged. Two ZDS proteins were identified; one had higher expression levels (1.4 (12 h), 1.7 (24 h), and 1.9 (48 h)), whereas the other had lower levels (0.8 (12 h), 0.9 (24 h), and 0.8 (48 h)), compared to those in the 0 h samples. In addition, the level of ABA2, which is involved in ABA biosynthesis, increased 1.6-fold after priming (12 h) and remained at this higher level.

#### Crucial DEGs involved in the volatile VPB biosynthesis pathway

Furthermore, we found 13 important DEPs (phenylalanine ammonia-lyases, PALs; beta-glucosidase; 4-coumarate-CoA ligase; cinnamoyl-CoA reductase, CCR; trans-cinnamate 4-monooxygenase, CYP73A; cinnamyl-alcohol dehydrogenase; coniferyl-alcohol glucosyltransferase, UGT72E; shikimate O-hydroxycinnamoyltransferase, HCT; coumaroylquinic (coumaroylshikimate) 3′-monooxygenase, CYP98A3; caffeoyl-CoA O-methyltransferase; glucosyltransferase; benzyl alcohol benzoyl transferase; BEBT; and salicylate O-methyltransferase, SOMT) involved in the phenylpropanoid biosynthesis pathway. PALs, which function as key enzymes in the core structure biosynthesis of VPBs, were highly abundant in the primed samples (1.2-1.4-fold increase). Additionally, the levels of cytochrome P450 enzymes, which contribute to hydroxylation and peroxidation reactions, exhibited significant changes. The levels of trans-cinnamate 4-monooxygenases were 1.5-, 1.6-, and 1.5-fold higher in the samples primed with MeJA for 12 h, 24 h, and 48 h, respectively, than in the untreated samples. The levels of CYP98A3 increased by 1.2-, 1.1-, and 1.2-fold, respectively. Additionally, proteins encoding enzymes modifying the basic structure of secondary metabolites, such as UDP-glycoside transferases and O-methyltransferases, including UGT72E, caffeoyl-CoA O-methyltransferase, salicylate O-methyltransferase, and several phenylpropanoid glucosyltransferases, showed differential abundance in the samples. To date, the precise biological function remains unclear, and the corresponding enzymatic characterization needs to be performed in subsequent studies. Moreover, BEBT, which belongs to the BAHD enzyme family and converts benzyl alcohol to benzyl benzoate, was found to be overexpressed in the treated samples (1.3-, 1.8-, and 1.4-fold) compared to the untreated samples.

Furthermore, DEPs were also characterized for several other pathways related to primary metabolism and precursor biosynthesis, such as amino acid metabolism and flavonoid and alkaloid metabolism.

### Differential metabolomic profiling of MeJA-primed tea leaves

We performed MS-based metabolomic analysis, which revealed a global set of plant metabolites, including volatile and nonvolatile metabolites, impacted by the application of MeJA to tea leaves. Multivariate statistical analyses were performed, including principal component analysis (PCA) and hierarchical clustering analysis (HCA) (Figs. S4 and S5). Significant differences among the MeJA-treated samples were observed at certain time points (0, 12, 24, and 48 h) at the metabolite level.

More than 2000 differential nonvolatile secondary metabolites were traced utilizing UPLC-Q-ToF/MS, and the differentially expressed metabolites were preliminarily identified and included fatty acid derivatives, phenylpropanoids, and TPs (Table S2). These results clearly indicate that differences in chemotypes depend on the MeJA priming duration (0, 12, 24, and 48 h). Two hundred and fifty-one metabolites were tentatively identified by combining reference information from the Metlin-plant and in-house databases. Additionally, 12 metabolites were accurately identified based on authentic reference compounds (Table S3; MSMS information was collected as shown in Fig. S6). All metabolites were assigned to seven secondary metabolic pathways, including the α-linolenic acid- (42), TP- (32), phenylpropanoid- (41), indole- (4), flavonoid- (118), carotenoid- (9), and amino acid-related (5) pathways. An additional eleven compounds could not be subclassified and formed the group of unknowns (Table S3).

Moreover, volatile metabolites were also identified based on GC-TQMS analysis, and 100 volatiles exhibited changes after MeJA priming. The volatiles were tentatively identified by the NIST and Wiley databases (Table S4). All of these volatile metabolites were classified into six secondary metabolic pathways, including the α-linolenic acid- (38), TP- (20), phenylpropanoid- (20), indole- (1), carotenoid-related (3) pathways, as well as benzoate degradation (2) and unknown (16) pathways. This result clearly demonstrates the complementary outcome of LC-MS- and GC-MS-based metabolomics and the importance of multiple mass spectrometric methods for the elucidation of complex metabolic changes.

### Quantification of five major carotenoids in MeJA-primed tea leaves

Quantitative analysis of the carotenoids present in the MeJA-primed tea leaves, including α-carotene, β-carotene, lutein, neoxanthin, and zeaxanthin, was performed. The total carotenoid content in MeJA-primed tea leaves showed a significant increase at 12 h (825.02 ± 1.95 µg g^−1^) and a dramatic decrease at subsequent time points (661.61 ± 1.9 (24 h) and 643.71 ± 1.9 (48 h) µg g^−1^) compared to the 0 h samples (780.35 ± 1.94 µg g^−1^), as shown in Table 1. Among the five quantified carotenoids, lutein demonstrated the highest percentage, greater than 72% on average, followed by β-carotene. The lutein content was higher in the treated samples at 12 h (596.62 ± 5.85 µg g^−1^) than in the other samples, while the α-carotenoid and neoxanthin levels showed a decreasing trend in the treated samples at 12 h.

**Table 1:** Absolute content of five major carotenoids identified using UHPLC-QToF/MS in MeJA-primed tea leaves

Samples	Concentration (µg g^−1^)					
	α-Carotene	Lutein	β-Carotene	Zeaxanthin	Neoxanthin	In total
MT_0 h	35.8 ± 0.35a	541.49 ± 5.31b	133.47 ± 1.31ab	24.64 ± 1.37ab	44.95 ± 1.46a	780.35 ± 1.94b
MT_12 h	28.63 ± 0.28b	596.62 ± 5.85a	137.38 ± 1.35a	26.27 ± 1.38a	36.13 ± 1.43b	825.02 ± 1.95a
MT_24 h	23.29 ± 0.23c	486.36 ± 4.77c	111.97 ± 1.1b	20.95 ± 1.35b	19.04 ± 1.34d	661.61 ± 1.9c
MT_48 h	16.98 ± 0.17d	470.91 ± 4.62c	109.67 ± 1.08b	23 ± 1.36ab	23.15 ± 1.37c	643.71 ± 1.9c

The different letters after the data within a column represent significant differences at the 95% probability level. The values are presented as the mean ± SD (µg g^−1^)

### Comprehensive understanding of MeJA-primed tea leaves by “multiomics” data integration and visualization

The “omics” strategy in this study offers large-scale data for integration into the elucidation of floral aroma biosynthesis pathways as well as the opportunity to elucidate dynamic changes in multiple dimensions after MeJA priming in *C. sinensis*. Key enzymes identified from the GLV, volatile TP, and VPB biosynthesis pathways were combined with the results for volatile and nonvolatile metabolites. All the data were integrated into self-organizing map-based pathways representing molecular signatures and corresponding chemotypes at different MeJA priming intervals. Color grading was used for visualization, with increases shown in red and decreases shown in green. Changes are given in relation to the control (0 h).

#### Consuming lipid precursors resulted in the release of GLVs and storage of glycosides

**Fig. 2:**
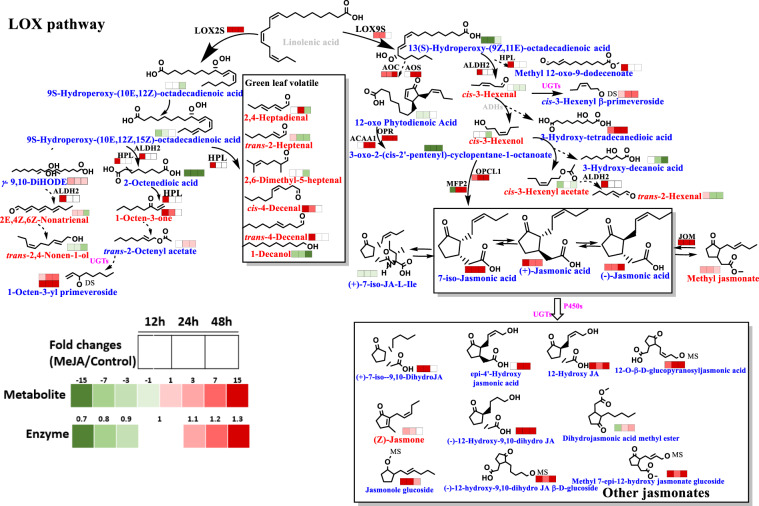
Response of green leaf volatile (GLV) biosynthesis in MeJA-primed fresh tea leaves. α-Linolenic acids serve as precursors for a variety of fatty acid-derived volatile organic compounds, also known as green leaf volatiles (GLVs). This precursor enters the lipoxygenase (LOX) pathway by oxidation, yielding 9-hydroperoxy and 13-hydroperoxy intermediates that are further converted to volatiles by hydroperoxide lyases and alcohol dehydrogenases. Differentially expressed enzymes and metabolites (volatiles shown in red letters; nonvolatiles shown in blue letters) were integrated into the pathway-based maps. Jasmonic acid metabolites are also displayed. Abbreviations are shown on the upper right of this figure. Red represents an increase, and green represents a decrease. Dashed arrows indicate unverified steps and include several steps in the pathway. Detailed fold changes are shown in Supplementary Tables S1, S2, and S4. LOX lipoxygenase, AOC allene oxide cyclase, JOM jasmonate O-methyltransferase, ADH alcohol dehydrogenase, HPL hydroperoxide lyase, UGT UDP-glycosyltransferase, OPR 12-oxophytodienoic acid reductase, ALDH2 aldehyde dehydrogenase, HPL hydroperoxide lyase, MFP2 enoyl-CoA hydratase, ACAA1 acetyl-CoA acyltransferase 1, AOS allene oxide synthase, OPCL1 OPC-8:0 CoA ligase 1, MS monosaccharide, DS disaccharide, g-9,10-DiHODE (+/-)-9,10-dihydroxy-6Z,12Z-octadecadienoic acid, (+)-7-iso-JA-L-Ile (+)-7-iso-jasmonic acid-L-isoleucine

MeJA priming induced the formation of GLVs from lipids and induced the storage of glycosides of GLVs as precursors. This could be associated with the higher abundance of LOXs (Fig. 2). It can be reasonably inferred that the dramatic decrease in 13S-hydroperoxy-(9*Z*,11*E*)-octadecadienoic acid (13*S*-HpODE) was due to its utilization as a direct precursor for successive C6–C9 GLV biosynthesis. High expression levels of AOS and AOC were involved in the first step of the catalytic conversion of 13S-HpODE to 12-oxophytodienoic acid (12-OPDA). Subsequently, validation of the higher expression levels of both 12-oxophytodienoate reductase (OPR) and acetyl-CoA acyltransferase 1 (ACAA1) indicated efficient conversion from 12-OPDA to 3-oxo-2-(cis-2’-pentenyl)-cyclopentane-1-octanoate. This is the first time that this crucial intermediate (3-oxo-2-(cis-2′-pentenyl)-cyclopentane-1-octanoate) has been tentatively identified in samples. Interestingly, the intermediate was continuously consumed, which resulted in the formation of a series of JA products. The expression of enoyl-CoA hydratase (MFP2) decreased in the treated samples at 12 h but increased at later time points. We hypothesize that MFP2 acts as a switch controlling JA biosynthesis and is also causally related to the difference observed after the different priming durations. Often, the results after 12 h of priming differ from those after 24 h and 48 h of priming, for instance, the expression levels of HPL and ALDH2. In the case of excess JA accumulation after priming, the expression of MFP2 was inhibited, leading to a slow down of further JA biosynthesis. However, after the conversion of JAs to JA derivatives, MFP2 was re-expressed to maintain its physiological levels. It is important to investigate the identified hydroxylated JAs in MeJA-primed leaves, especially 12-hydroxy- and 4′-hydroxy-JAs, which have been reported to deactivate the JA pathway in tea plants.

In our study, the (*Z*)-3-hexenal, (*E*)-2-hexenal, and (*Z*)-3-hexenyl acetate levels showed a decreasing trend in MeJA-primed tea leaves. It is possible that priming activated a direct defense strategy by releasing large amounts of GLVs. In addition, plants respond via the formation of glycosidic GLVs (hexenyl glycosides, 1-octen-3-yl glycosides) and contribute to the decrease in free-form GLVs.

### Accumulation of volatile TPs and the related glycosidically bound precursor

**Fig. 3:**
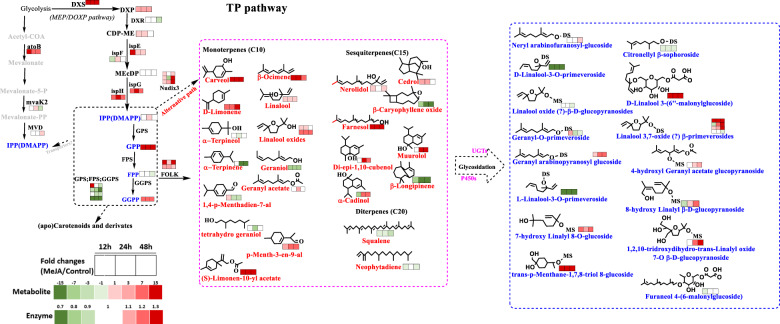
Volatile terpenoid (TP) biosynthesis in MeJA-primed fresh tea leaves. Terpenoid volatile organic compounds are synthesized by the cytosolic mevalonic acid (MVA) and plastidial methylerythritol phosphate (MEP) pathways. Differentially expressed enzymes and metabolites were integrated into pathway-based maps. Mono-, sesqui-, and diterpenes are shown in the pink dashed box, and glycosidically bound derivatives are shown in the blue dashed box. Carotenoids and their derivatives were also derived through the pathway and map shown in the next figure. Cross-talk between both pathways is facilitated by the export of isopentenyl pyrophosphate (IPP) from the plastid to the cytosol. TPs terpenoids, CDP-ME 4-(cytidine 5′-diphospho)-2-C-methyl-D-erythritol, MEcDP 2-C-methyl-D-erythritol 2,4-cyclodiphosphate, IPP isopentenyl pyrophosphate, DMAPP dimethylallyl pyrophosphate, GPP geranyl diphosphate, FPP farnesyl diphosphate, GGPP geranyl geranyl diphosphate, DXS 1-deoxy-D-xylulose-5-phosphate synthase, DXR 1-deoxy-D-xylulose 5-phosphate reductoisomerase, atoB acetyl-CoA C-acetyltransferase, mvaK2 phosphomevalonate kinase, MVD diphosphomevalonate decarboxylase, ispE 4-diphosphocytidyl-2-C-methyl-D-erythritol kinase, ispF 2-C-methyl-D-erythritol 2,4-cyclodiphosphate synthase, ispG (*E*)-4-hydroxy-3-methylbut-2-enyl-diphosphate synthase, ispH 4-hydroxy-3-methylbut-2-enyl diphosphate reductase, GPS geranyl diphosphate synthase, FPS farnesyl diphosphate synthase, GGPS geranyl geranyl diphosphate synthase, FOLK farnesol kinase

The plastidial methylerythritol phosphate (MEP) branch responded strongly after MeJA treatment (Fig. 3). DXS, DXR, ispE, ispG, ispH, GPS, FPS, and GGPS were differentially expressed in response to priming, and they all showed much higher expression in the treated samples at 12 h and 24 h than in the untreated samples. This finding is supported by the changes in intermediate metabolites, including DXP and CDP-ME, and identified volatile TP precursors, such as GPP and GGPP. The levels of all of these metabolites remained consistent with the levels of their upstream proteins. The only exceptions were ispF and its downstream intermediate MEcDP, the expression of which was significantly downregulated in the samples primed with MeJA for 12 h and 24 h. We hypothesized that the aforementioned enzyme and intermediate represented a key restricted metabolism point in the MEP branch when tea plants were exposed to biotic stress; however, this hypothesis needs further investigation.

Priming induced the production of floral TPs, e.g., linalool and its oxides, nerolidol, limonene, and others. In contrast, for instance, the levels of geraniol, β-caryophyllene oxide, and β-longipinene decreased in response to priming after 12 h. Although significant differences were observed at the metabolomic level, no apparent changes in the expression levels of terpene synthase (TPS) were found in MeJA-primed leaves compared to untreated leaves. A possible explanation might be that these changes occurred earlier after MeJA priming. Interestingly, three nudix enzymes, which possibly catalyze the conversion of GPP to GP, might also be effective in geraniol biosynthesis via phosphatase activity. These proteins showed significantly increased expression in the treated samples at 12 h, a slight decrease at 24 h, and then a significant increase again at 48 h. Further experiments need to be well scheduled.

**Fig. 4:**
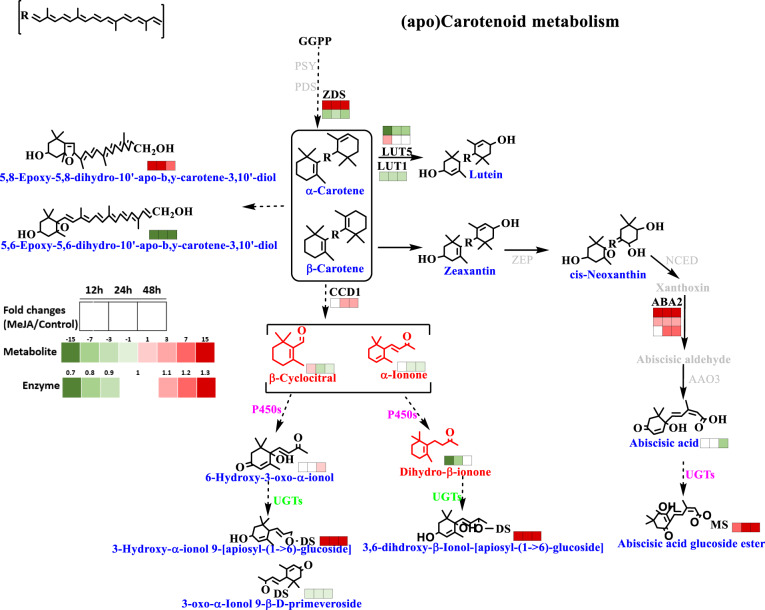
Metabolism of carotenoids and their derivatives in MeJA-primed fresh tea leaves. Carotenoids and their derivatives were directly formed from the TP pathway. Differentially expressed enzymes and metabolites included were integrated into pathway-based maps. The five major carotenoids, including α-carotene, β-carotene, lutein, neoxanthin, and zeaxanthin, were identified, and the absolute quantification results are listed in Table 1. ZDS zeta-carotene desaturase, CCD1 carotenoid cleavage dioxygenase, NCED 9-cis-epoxycarotenoid dioxygenase, b-LCY lycopene beta-cyclase, PSY phytoene synthase, ZEP zeaxanthin epoxidase, PDS 15-cis-phytoene desaturase, beta-carotene 3-hydroxylase, LUT5/LUT1 β-carotene hydroxylase, ABA2 xanthoxin dehydrogenase, AAO3 abscisic-aldehyde oxidase

Carotenoids and their derivatives are directly formed within the TP pathway. There have been a few reports on the response of this branch to MeJA priming. Differentially expressed enzymes and metabolites were integrated into the pathway-based map, as shown in Fig. 4. Compared with untreated samples, the treated samples showed an increase in the total carotenoid content (Table 1); in particular, large amounts of β-carotene, lutein, and zeaxanthin were accumulated in the treated fresh leaf samples at 12 h (Fig. 4). The level of CCD1 showed significant changes in response to different treatment durations. The enzymatic products of CCDs, especially citral and ionone, were subjected to dynamic changes and indicate that basal carotenoid catabolism and metabolic changes induced by priming occur simultaneously.

Compared to GLVs, volatile TPs are characterized by lower volatility, and the widely biosynthesized TPs are further stabilized via the formation of their glycosides. In plants, volatile TPs and their derivatives are mainly present in glycosidically bound forms; in this study, neryl glycosides, citronellyl glycosides, geranyl glycosides, 7-hydroxyl linalyl glycosides, 4-hydroxyl geranyl acetate glycosides, 3-hydroxyl-α-ionol glycoside and 3,6-dihydroxyl-β-ionol glycoside, which are part of the TP pathway, were found to accumulate in MeJA-primed tea leaves.

### Changes in VPBs and their corresponding precursors

**Fig. 5:**
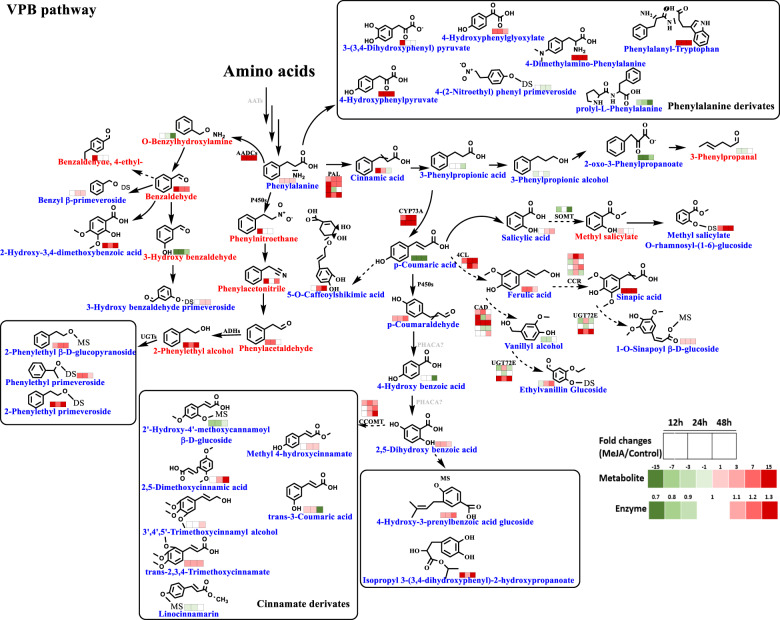
Volatile phenylpropanoid/benzenoid (VPB) biosynthesis in MeJA-primed fresh tea leaves. Phenylpropanoid and benzenoid volatiles are invariably derived from the common precursor phenylalanine, which is synthesized in plastids through shikimate/phenylalanine biosynthetic pathways. The differentially expressed enzymes and metabolites included were integrated into pathway-based maps. ADHs alcohol dehydrogenases, P450s cytochrome P450s, UGT UDP-glycosyltransferase, MTs O-methyltransferases, ? unknown enzyme, PAL phenylalanine lyase, AADCs aromatic l-amino acid decarboxylases, PHACA phenylacetate 2-hydroxylase, CYP73A trans-cinnamate 4-monooxygenase, 4CL 4-coumarate--CoA ligase, CCOMT caffeoyl-CoA O-methyltransferase, CCR cinnamoyl-CoA reductase, CAD cinnamyl-alcohol dehydrogenase, UGT72E coniferyl-alcohol glucosyltransferase, HCT shikimate O-hydroxycinnamoyltransferase, SOMT salicylate O-methyltransferase-like

In terms of the complexity of floral compound metabolism in tea, the VPB pathway is most notable. The levels of benzaldehyde, phenylacetaldehyde, and phenyl alcohol, as very important aroma flavor contributors in tea, were observed to be significantly changed in tea leaves primed with MeJA (Fig. 5). O-benzylhydroxylamine was inferred to be the key intermediate catalyzed by certain enzymes and then further converted to benzaldehyde. Similarly, it could be concluded that phenylnitromethane is the precursor of phenylacetonitrile. In the adjacent branch, phenylacetaldehyde and 2-phenyl alcohol were observed to accumulate throughout the MeJA treatment period (12–48 h). Furthermore, we observed that the methyl salicylate levels increased and p-coumaraldehyde levels decreased significantly at 12 h; the former was catalyzed to its glycosidically bound form, while the latter was utilized as a precursor for other benzyl propanoids. The levels of cinnamic acid, p-coumaric acid, caffeic acid, ferulic acid, sinapic acid, and hydroxylated benzoic acid were all found to significantly change in response to MeJA priming (Fig. 5).

We also discovered numerous other key enzymes and the related changes that modulate the VPB pathway, including 4CL, CCR, CAD, and AADCs. Notably, in the VPB pathway, o-methyltransferases are important enzymes that result in the production of various methyl derivatives. We found that the expression of SOMT was downregulated during the treatment period; however, the expression of its upstream substrate (salicylic acid) and downstream product (methyl salicylate) was increased. In addition, the level of CCOMT, which catalyzes the conversion of cinnamic acid and other metabolites to their methylated forms, showed a definite increase (the relative content is marked in the box labeled ‘Cinnamate derivatives’). In addition, glycosides of 2-phenylethanol, benzyl aldehyde, 3-hydroxybenzaldehyde, and methyl salicylate accumulated in MeJA-primed tea leaves (Fig. 5). The biological importance and the mechanism by which the levels of free-form volatile metabolites and their glycosidic forms are balanced in fresh tea leaves require further in-depth research.

## Discussion

We combined proteomic and metabolomic analyses and comprehensively revealed the protein-to-metabolite changes that occur in MeJA-primed tea leaves. The integration of “omics” data into self-constructed maps offered clear visualization of key changes in three major volatile biosynthesis pathways that have a strong connection with tea aroma quality.

### JA pathway activation after MeJA priming

The α-linolenic acid degradation pathway is a rapid defense pathway that responds to exogenous elicitors^28^. In response to MeJA priming, the relative content of GLV C6 compounds decreased, and C8 (1-octen-3-one) and C9 (decanal and its derivatives) compounds accumulated. C8 and C9 derivatives exhibit antibacterial/viral/fungal properties^29^ or are released as defense compounds after insect damage. This phenomenon is caused by the induction of key LOX enzymes due to MeJA priming mimicking herbivorous insects^1^ and is commonly activated by jasmonate-dependent defense mechanisms. This timely response triggers the formation of fatty acid-derived volatiles in fresh tea leaves, which are eventually stored as glycosides and in turn improve the sensory properties of tea products^26^. Hence, MeJA priming could be used to activate defense mechanisms and reduce the amount of pesticide used during tea farming.

The OPR gene was recently identified, and the enzyme was cloned and characterized^17^. In this study, we annotated a strong OPR response after MeJA priming for 12 h. To the best of our knowledge, the present study is novel in that it reports the differential expression of the JMT protein in tea. According to other researchers^30^, JMT is a key enzyme involved in JA-regulated plant responses and the transfer of methyl groups to JA. The gene sequences encoding the two potential JMTs identified in this study were identical to the published sequence from *C. sinensis* (TSA: *Camellia sinensis* tea_rep_c7003 mRNA sequence; TSA: *Camellia sinensis* tea_c5935 mRNA sequence)^16,17,27,30^. Blasting against protein sequences from tea plants showed that the two potential JMTs had high similarity, especially JMT2, with >92% identity. These high similarities support the finding that JMTs also contribute to the accumulation of MeJA in this study. Mediation of free JA levels through conjugation has been discussed previously^31^. It is clear that 7-iso-JA and its isoleucine conjugates are core compounds that activate the whole JA pathway in response to stress or herbivore attack^1,32^. However, these compounds do not easily shuttle between lipid-based membranes. This has already been verified in *Arabidopsis* and *Solanaceae*, in which MeJA is transported and essentially metabolized to its active form JA-Ile. Consequently, the entry and exit of MeJA into and out of cells is fundamental to whole-plant JA pathway activation. The flux and dynamic mediation of free JA levels through conjugation highlight the biological role of JA metabolism as the foundation for the improvement of tea flavor due to MeJA priming. This provides evidence that MeJA acts as a transportable intercellular mobile compound in tea plants that triggers whole-plant secondary metabolite dynamics.

### Metabolite dynamics in response to MeJA-priming

GLVs were biosynthesized in large amounts in response to priming and were successively released into the surroundings. The JA pathway, activated to a great degree by plants entering defense mode, can be excessively depleted by GLVs^33^. However, plants are self-balancing experts and have developed an adaptation strategy. Other studies have shown that activated JA pathways can slow down or even shut down after JAs are metabolized, mainly to hydroxylated and glycosylated compounds. As previously reported^20,33^, cytochrome P450s are easily affected by exogenous stimuli and are associated with detoxification mechanisms, including the release of volatiles. The large-scale biosynthesis of 12-hydroxy- and 4′-hydroxy-JAs in tea leaves is also an effective strategy to balance the defense response and for regular adaptation. In addition, the accumulation of hydroxylated JA glycosides appears to be another strategy; however, to date, the mechanism has not been determined. Glycosylation conjugates secondary metabolites in plants, which facilitates the storage and transport of hydrophobic substances and reduces their activity by blocking reactive hydroxyl groups^34,35^. UGTs are key for the conversion of secondary metabolites to various glycosides. Overall, based on our results, we hypothesized that hydroxylation and glycosylation were spontaneously initiated by MeJA priming and that the pathways remained activated during the experimental period.

### Potential promotion of aroma quality

As we have already acknowledged, GLVs (methyl jasmonate, C6–C9 volatiles), volatile TPs (linalool and its oxides, geraniol, nerolidol, citral, and ionone), and VPBs (benzyl alcohol, benzaldehyde, 2-phenylethanol, and phenylacetaldehyde) are the most important aroma compounds in tea and mostly contribute a floral and sweet flavor^36,37^. These volatile classes were enriched after priming. This resulted in better tea quality and higher market value. As previously reported^36^, excessive volatiles stored in their free form in plants can, to a large extent, cause damage; thus, glycosylation is the effective form of detoxification. Twenty-seven volatile glycosides were stably stored, 3 were involved in GLV pathways, 15 were involved in the TP biosynthesis pathway, 3 were involved in the carotenoid metabolism pathway, and 7 were involved in the VPB biosynthesis pathway. According to other studies^38–40^, glycosides exhibit very stable chemical and structural behaviors that promote their long-term storage in plants. Free volatile TPs and VPBs accumulate in fresh leaves but are mainly lost during tea manufacturing. The storage of volatile glycosides is stable in fresh tea leaves, and they can be released in their free form via either enzymatic (withering, rolling, fermentation, etc.) or nonenzymatic (fixation, drying) processes during tea manufacturing^40–42^. In this study, we identified several UDP-glycosyltransferases (Table S1) that likely contribute to the release of volatiles from their glycosidic precursors. The different activities of these glycosyltransferases toward different aroma substrates could be a major reason for the accumulation of various glycosides in MeJA-primed tea leaves, which was also confirmed by another study^35^. In addition, various families of UGTs, which play key roles in the glycosylation of phenolic compounds, were identified^43–45^. Further characterization of UGTs will shed more light on the mechanisms triggering the release of volatiles and the accumulation of glycosidically bound precursors.

In conclusion, MeJA, an elicitor in plants, was effectively primed in tea plants, resulting in a significant change at the protein and metabolite levels. Multiomic analysis revealed that the GLV biosynthesis pathway responded quickly to exogenous MeJA priming, and simultaneously, the TP and VPB biosynthesis pathways were promoted. These changes together with the accumulation of aroma glycosides are the key regulators of the production of higher-quality tea products from MeJA-primed tea leaves.

## Materials and methods

### Tea plants and methyl jasmonate priming

Two-year-old plants of Jinxuan, a cultivar of tea (*C. sinensis*), were planted in the greenhouse of the Tea Research Institute, Chinese Academy of Agricultural Sciences, Hangzhou, Zhejiang Province, P.R. China. Samples were treated and prepared in spring (early April). A thousand tea plants individually planted in an isolated chamber were evenly sprayed with 8 L of a 0.25% (v/v) aqueous solution of MeJA, which was pre-dissolved in 200 mL of ethanol. Two fresh tea leaves and one bud were plucked at 0, 12, 24, and 48 h after treatment. The plucked tea leaves were immediately frozen in liquid nitrogen for subsequent experiments, including proteomics and metabolomics.

To minimize biological variance, each sample was harvested in three independent biological replicates of at least 300 g of tea leaves and subsequently pooled for all analyses. iTRAQ-based proteomics and MS-based secondary metabolite analysis were performed in at least three replicates.

### Chemicals and reagents

The standards listed in Table S3 were all purchased from Sigma (Sigma-Aldrich, Germany). Other chemicals/biochemicals and reagents used for the analyses were also purchased from Sigma unless otherwise noted.

### Proteomic analysis and data reorganization

iTRAQ-based proteomic analysis, including protein extraction and preparation, iTRAQ labeling and strong cation exchange, LC-ESI-MS/MS analysis, and protein identification and annotation, was performed by BGI Genomics Co., Ltd. (Shenzhen China), and the detailed procedures are listed in the Supplementary materials. The data were reorganized and evaluated by us.

The LC/ESI-MS/MS data were analyzed using Proteome Discoverer 1.3 software (Thermo Fisher Scientific, San Jose, CA, USA) with default parameters to generate a peak list. Proteins were identified using the Mascot search engine (version 2.3.02) (Matrix Science, London, UK) against the NCBI_TSA: *C. sinensis* unigenes protein database (nr, 922806) (https://www.ncbi.nlm.nih.gov/nuccore/?term=(TSA)%20AND%20%22Camellia%20sinensis%22[porgn:__txid4442]). The Mascot search engine uses probability-based scoring to determine whether results are significant (www.matrixscience.com/help/scoring_help.html#PBM). Only peptides with a confidence interval ≥95% according to their Mascot probability scores were considered to have been identified. Protein quantitation was performed at the peptide level following an established procedure (http://www.matrixscience.com/help/quant_format_help.html). For quantification, proteins with at least one unique peptide and an unused value greater than 1.3 (credibility ≥ 95%) were considered for further analysis. The manufacturer’s recommended isotope correction factors were applied. We only used ratios with *p* values <0.05, and ratios >1.25 or <0.85 were considered significant. GO was used to functionally classify the differentially expressed proteins (DEPs) (http://www.geneontology.org). COG is a database for classifying protein orthologs and was also used to evaluate potential protein functions. The Kyoto Encyclopedia of Genes and Genomes (KEGG) database (http://www.genome.jp/kegg/) was used to clarify the pathways that the DEPs may be associated with.

### Volatile metabolite characterization by GC-TQMS analysis

Stir bar sorptive extraction devices (GERSTEL-Twisters, polydimethylsiloxane phase, Gerstel GmbH & Co. KG, Germany) were used for volatile enrichment. Fifty milligrams of lyophilized tea sample was weighed into a 50 mL vial, and 10 mL of 100 °C preheated distilled water was successively added to the twisters. The vials were placed into a water bath at 60 °C, and the extraction lasted for 45 min. Twisters were washed twice with ultrapure water, dried with lint-free tissues and placed into 2 mL GC vials until further analysis.

Volatiles were analyzed with an Agilent 7890B gas chromatography and an Agilent 7010 GC/MS Triple Quad. The gas chromatography was equipped with a BP5MS column (30 m × 250 μm i.d., 0.25 μm; SGE Analytical Sciences). GC-MS with a Gerstel MPS 2 (multiple purpose sampler) injection system was performed with the following oven temperature program: 40 °C for 3 min, increase of 2 °C/min until 60 °C, hold for 2 min, increase of 3 °C/min until 180 °C, and hold for 10 min isothermally. The carrier gas used was helium and was maintained at a constant flow rate of 1.2 mL/min. Twister desorption was performed with a Gerstel Thermal Desorption Unit with the following temperature program (starting temperature, 25 °C; increase of 100 °C/min until 250 °C; hold for 4 min at 250 °C). The cryofocusing program started at −100 °C. The volatiles were released and transferred to the GC by increasing the temperature at 12 °C/min to 280 °C and then maintaining the temperature at 280 °C for 3 min. The MS analysis was carried out in full-scan mode, with a scan range of *m/z* 50−300. The electron impact ionization energy was 70 eV for all measurements. Compounds were identified tentatively by comparing the mass spectra with the Wiley 6.L and NIST 98.L libraries. The raw data were obtained after automatic and manual integration of the peak area using MassHunter (version B. 07.00; Agilent Technologies).

### Nonvolatile metabolite characterization based on UHPLC-QToF/MS analysis

Then, 1.5 mL of methanol/water solution (70/30, v/v) containing 0.1% formic acid was added to 200 mg of ground tea powder, and extraction was performed by ultrasonication for 10 min. After centrifugation at 8500 × *g* (Centrifuge 5810R, Eppendorf) for 10 min, the supernatants were transferred to a 10 mL volumetric flask. The steps were repeated five times. Next, the volume was made up to 10 mL with extraction solvent. An aliquot was filtered through a 0.22 μm nylon membrane (TEFIC, Shanxi, China), and the filtrate was transferred to an HPLC vial.

Quality control (QC) samples were prepared by mixing equal volumes of each tea sample. The metabolomic measurements were carried out on an ultrahigh-performance liquid chromatography system (UHPLC Infinity 1290, Agilent Tech, USA) coupled to a quadrupole-time-of-flight mass spectrometer (Q-ToF 6540, Agilent Tech, Santa Clara, CA). Chromatographic separation was performed on a Zorbax Eclipse Plus C18 column (100 × 2.1 mm, 1.8 μm, Agilent Tech, Littlefalls, DE) at a constant column temperature of 40 °C. Binary mobile phases were used for gradient elution through online mixing of solvent A and solvent B, wherein solvent A was an aqueous solution containing 5 mmol/L ammonium acetate and 0.1% (v/v) formic acid and solvent B was a methanol solution containing 5 mmol/L ammonium acetate and 0.1% (v/v) formic acid. The linear gradient program was as follows: 0 min, 10% B; 4 min, 15% B; 7 min, 25% B; 9 min, 32% B; 16 min, 40% B; 22 min, 55% B; 28 min, 95% B; 30 min, 95% B. The total elapsed time required for a given chromatographic analysis was thus 30 min. Subsequently, 4 min was required for equilibration of the column between two consecutive injections. The flow rate was set at 0.4 mL/min. The injection volume was 1 μL. The eluent from the column was directed to a dual-jet stream electrospray ionization (ESI) source interfaced with the Q-ToF mass spectrometer and operated in negative mode with the following parameters: capillary voltage set at 3500 V; temperature and flow rate of drying gas set at 300 °C and 8 L/min, respectively; nebulizer pressure set at 35 psi; temperature and flow rate of the sheath gas maintained at 300 °C and 11 L/min, respectively; and a mass scan range of 100–1100 *m/z* applied for the full-scan analysis. The Q-ToF/MS was calibrated daily following the manufacturer’s procedure, and reference ions with *m/z* values of 121.0509 (purine) and 922.0098 (hexakis phosphazine) were infused by a reference sprayer during data acquisition for online calibration, ensuring MS accuracy within 3 ppm.

### Data analysis and multivariate statistics

The raw data from the GC analysis were deconvoluted and processed by Mass Profiler Professional (MPP; Version 12.1, Agilent, Technologies, Santa Clara, CA, USA). Compounds with a minimum abundance of 70% in all samples of one treatment were subjected to statistical analysis, which included one-way ANOVA followed by Tukey’s honestly significant difference (HSD) post hoc test (*p* < 0.01; fold change ≥ 2). The UPLC-ToF/MS data were subjected to the recursive workflow by Mass Hunter qualitative analysis and MPP, including peak picking, alignment of detected features, integration, and peak area calculation.

Each sample was normalized to the median of the baseline and log2 transformed. Compounds with a minimum abundance of 70% in all samples of one treatment were subjected to statistical analysis. One-way ANOVA (*p* < 0.01; fold change ≥ 2) followed by Tukey’s honestly significant difference (HSD) post hoc test (*p* < 0.01; fold change ≥ 2) was performed to identify the significantly different features. Differential compounds were tentatively identified using Mass Hunter Metlin PCD (version 4.0, 24768 compounds). Multivariate data analyses were carried out by MPP using the dataset identifying significant differentially abundant compounds.

### Targeted analysis of carotenoids using UHPLC-QToF/MS

Five milligrams of finely ground tea samples was extracted by shaking with 500 µL of MeOH/THF (1:1, v/v) solution for 5 min at 1500 × *g* and 20 °C. After centrifugation at 4800 × *g* for 5 min at 20 °C, the supernatant was transferred to a 4 mL glass vial. The extraction was repeated three times. The combined supernatants were evaporated to dryness in a stream of nitrogen. Prior to UHPLC analysis, the samples were dissolved first in 50 µL of DCM and subsequently in 200 µL of isopropanol by ultrasonication. The solution was filtered using a polytetrafluoroethylene (PTFE) centrifuge filter for 2 min at 3200 × *g* and 20 °C. The filtrate was transferred to a vial with an insert, and the sample protected in a nitrogen atmosphere.

Separation was performed on a YMC C30 column (100 × 2.1 mm, 3 µm; YMC Co., Ltd., Japan) using an Agilent Technologies 1290 Infinity ultrahigh-performance liquid chromatograph. Prior to analysis, solutions were filtered through a 0.2 μM PTFE membrane and kept at 4 °C in the autosampler during the analysis. Mixtures of methanol, methyl-tert-butyl ether, and water in different volumetric ratios (solvent A = 81/15/4 and solvent B = 6/90/4) were used as mobile phases at a flow rate of 0.2 mL/min. Carotenoids were separated in gradient mode from 100% (10 min isocratic) to 0% solvent A within 60 min. To enhance ionization, 20 mM ammonium acetate was added to the mobile phases. Pigments were analyzed on an Agilent Technologies 6230 time-of-flight (ToF) LC/MS equipped with an atmospheric-pressure chemical ionization ion source in positive ionization mode. The gas temperature was set to 325 °C, the gas flow rate was set to 8 L/min, the vaporizer was set to 350 °C, and the nebulizer pressure was set to 35 psi. The voltage was set to 3500 V, and a fragmentor voltage of 175 V was applied with a corona current of 6.5 μA. Carotenoid standards were prepared, and concentrations were determined spectrophotometrically using the specific wavelengths and extinction coefficients^46^. Compounds were identified by cochromatography with reference substances using the (all-*E*)-isomers for β-carotene, lutein, zeaxanthin, and neoxanthin. External standard calibration curves were used for quantification by dose-response curves, and quantification was performed at a detection wavelength of 450 nm. The data analysis was carried out by using Mass Hunter ToF Quantitative Analysis (version B 05.00, Agilent Technologies) using external calibration curves.

### Multiomic integration and pathway visualization

Integration of proteomic and metabolomic data, focusing mainly on GLV, volatile TP and VPB biosynthesis pathways, was performed by referring to the Kyoto Encyclopedia of Genes and Genomes (KEGG) maps. The pathway-based maps were customized and visualized using ChemDraw Std (version 13.0).

## Supplementary information

SuppIemental materials

Table S1. Differential expressed proteins

Table S2. Differential nonvolatile metabolites

Table S3. Information of standards

Table S4. Differential volatile metabolites
