# Supplementary material for: Integrated proteomic and metabolomic analyses reveal the importance of aroma precursor accumulation and storage in methyl jasmonate-primed tea leaves
Source: Hortic Res. 2021 May 1;8:95. doi: 10.1038/s41438-021-00528-9 (PMC8087812; doi:10.1038/s41438-021-00528-9)
Supplement: Supplementary file 1 — SuppIemental materials [file 41438_2021_528_MOESM1_ESM.docx]

## SUPPORTING INFORMATION

**SUPPLEMENTARY METHODS**

**Detailed methods involved in proteomics:**

**Protein extraction and preparation**

Initially, MeJA-primed tea leaves samples (approximately 500 mg of each portion) were ground to a fine powder in liquid nitrogen. Then treated with lysis buffer (7 M urea, 2 M thiourea, 4% 3-[(3-cholamidopropyl)dimethylammonio]propanesulfonate (CHAPS), 40 mM Tris-HCl, pH 8.5) containing 1 mM phenylmethylsulfonyl fluoride (PMSF) and 2 mM ethylene diamine tetraacetic acid (EDTA). After 5 min, 10 mM (final concentration) dithiothreitol (DTT) was added. The suspensions were all kept on the ice and sonicated for 15 min, and then centrifuged at 25,000 *g* (4°C) for 20 min. Supernatants were transferred to a new tube and treated with 5-fold volume of pre-cooled acetone in relation to the supernatant volume. Proteins were precipitated at -20°C for 2 h then centrifuged at 16,000 *g* (4°C) for 20 min and the supernatant transferred to a collection tube. Subsequently, the steps were repeated. The combined supernatants were incubated at 56°C for 1 h and then treated with 55 mM iodoacetamide (IAM) for 45 min in darkness. Supernatants were thoroughly mixed with pre-cooled acetone for 2 h at -20°C and then centrifuged at 25,000 *g* (4°C) for 20 min. And supernatants were discarded. Pellets were air-dried for 5 min before being dissolved in 500 μL 0.5 M tetraethylammonium bromide (TEAB) and sonicated at 200 W for 15 min. Samples were centrifuged at 25,000 *g* (4°C) for 20 min and then stored at -80°C for further analysis.

**iTRAQ labeling and strong cation exchange**

Total protein (100 μg) from each sample solution was digested with Trypsin Gold (Promega, Madison, WI, USA) (protein to trypsin weight ratio of 20:1) for 4 h at 37°C. Same amount Trypsin was added, for another 8h digestion at 37°C. Peptides were dried by vacuum centrifugation and then reconstituted and processed in 0.5 M TEAB according to the manufacturer’s protocol for 4-plex iTraq analysis (Applied Biosystems). The samples namely control (0 h), 12 h, 24 h, and 48 h after priming with MeJA, were labeled with different label tags, placed at ambient temperature for 2 h. All samples were mixed and then fractionated using an Ultremex SCX (strong cation exchange) column (4.6 × 250 mm) and the Shimadzu LC-20AB HPLC system (Shimadzu, Kyoto, Japan). The mixtures were dissolved with 4 mL buffer A (25 mM NaH2PO4 in 25% acetonitrile, pH 2.7) and loaded onto a SCX column containing 5-μm particles (Phenomenex) and eluted (1 mL/min) with buffer A for 10 min, followed by 5%-60% buffer B gradient (25 mM NaH2PO4, 1 M KCl in 25% acetonitrile, pH 2.7) for 25 min, and then a 60-100% buffer B gradient for 2 min. The system was then maintained at 100% buffer B for 1min. Eluted samples were quantified by absorbance at 214 nm. Finally, peptides were pooled into 20 fractions for LC-ESI-MS/MS analysis.

**LC-ESI-MS/MS analysis**

The LC-ESI-MS/MS analysis was based on the Triple TOF 5600 system (AB SCIEX, Concord, ON) with a Nanospray III source (AB SCIEX, Concord, ON). The 20 fractions were solubilized in buffer A (5% acetonitrile and 1% formic acid in ultrapure water) at a final concentration of 0.5 μg/μL. Samples of 5 μL (2.5 μg) were loaded onto an LC-20AD nano HPLC (Shimadzu, Kyoto, Japan) with the autosampler (2 cm C18 trap column). Then, peptides were eluted and loaded onto a C18 column (10 cm in length, inner diameter 75 μm, packed in-house) at a flow rate of 8 μL/min for 4 min and chromatographed for 35 min using a gradient of 2 to 35% buffer B (95% acetonitrile and 1% formic acid) at a flow rate of 300 nL/min, followed by ramping up to 60% buffer B within 5 min, up to 80% within2 min, maintained isocratic for 2 min, and returned to 5% buffer B in 1 min. Mass spectrometric analysis was depended on a data-dependent manner with full scans of the Orbitrap mass analyzer ( resolution:≥30 000 at m/z 400; automatic gain control: 500000 ions). The 30 most intense precursor ions were used for MS/MS fragmentation and detected at a mass resolution of 15000 at m/z 400 with peptides above a 5-count threshold selected and excluded for 30 s of 30 mDa mass tolerance. The fragmentation was activated with higher energy collision dissociation. Full Fourier transform mass spectrometry and MS/MS was set to 1 and 0.1 million ions, with a maximum time of accumulation of 2 s.

The general workflow of the iTRAQ experiment is presented in Fig. S1.

**MT_0**

**MT_12**

**MT_24**

**MT_48**


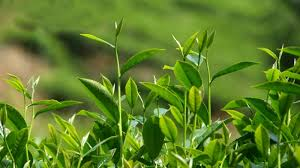


**Methyl Jasmonate**

**Protein extraction and preparation**

**Reduction, alkylation, trypsin digestion**

**iTRAQ**

labeling

114

**iTRAQ**

labeling

118

**iTRAQ**

labeling

119

**iTRAQ**

labeling

121

**Pool fraction(4.6x250mmUltremexSCX coloum )**

**LC-ESI-MSMS(Triple TOF 5600)**

**Protein identification and quantification**

**Fig. S1.** Workflow for the analysis of protein expression involving iTraq labeling in MeJA-primed fresh tea leaves with 0, 12, 24, 48 h duration


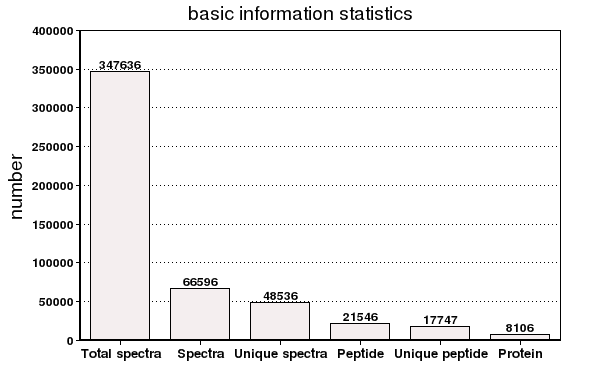

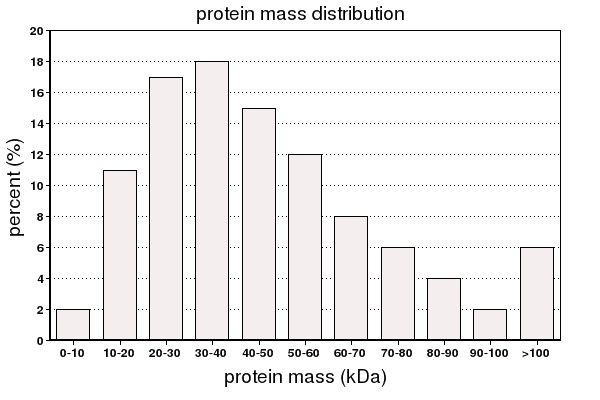

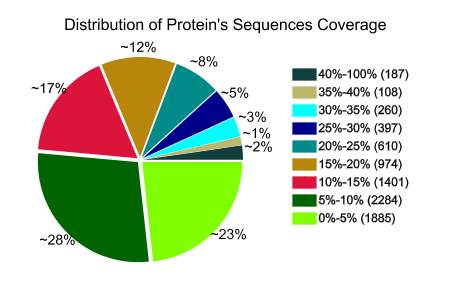


**B**

**C**

**A**

**Fig. S2.** Results of the iTraq liquid chromatography/tandem mass spectrometry analysis of MeJA-primed tea leaves.

**A**


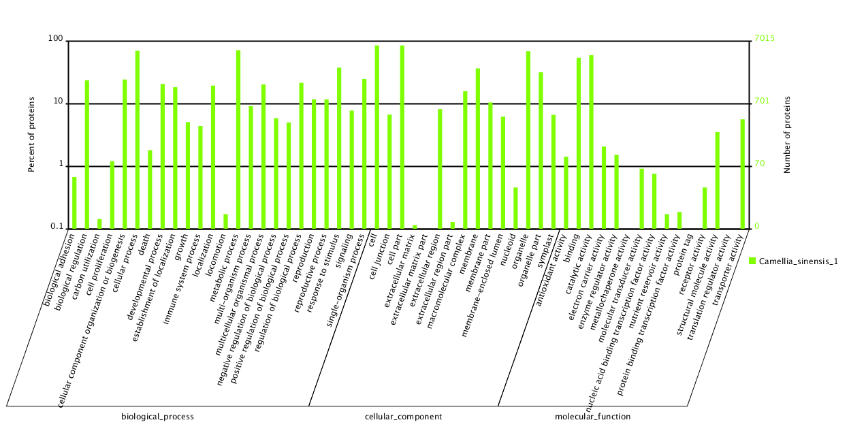


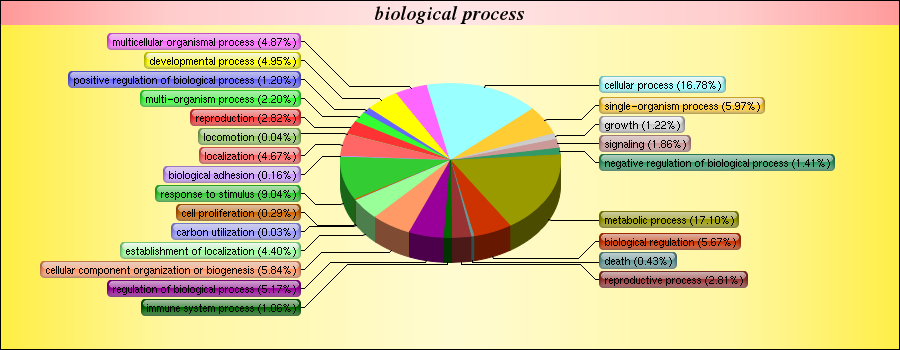


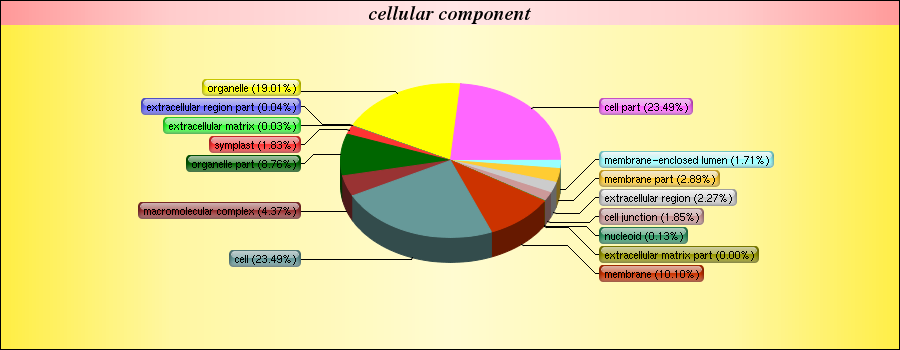


**
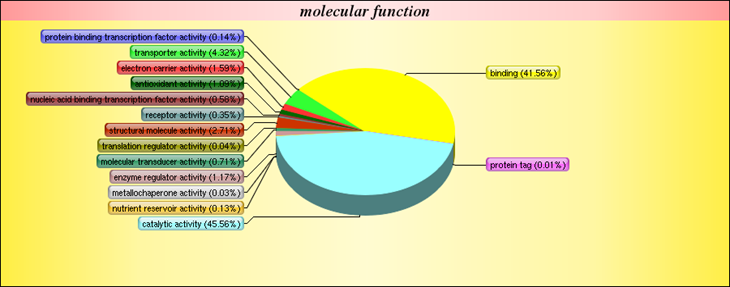
**

**B**


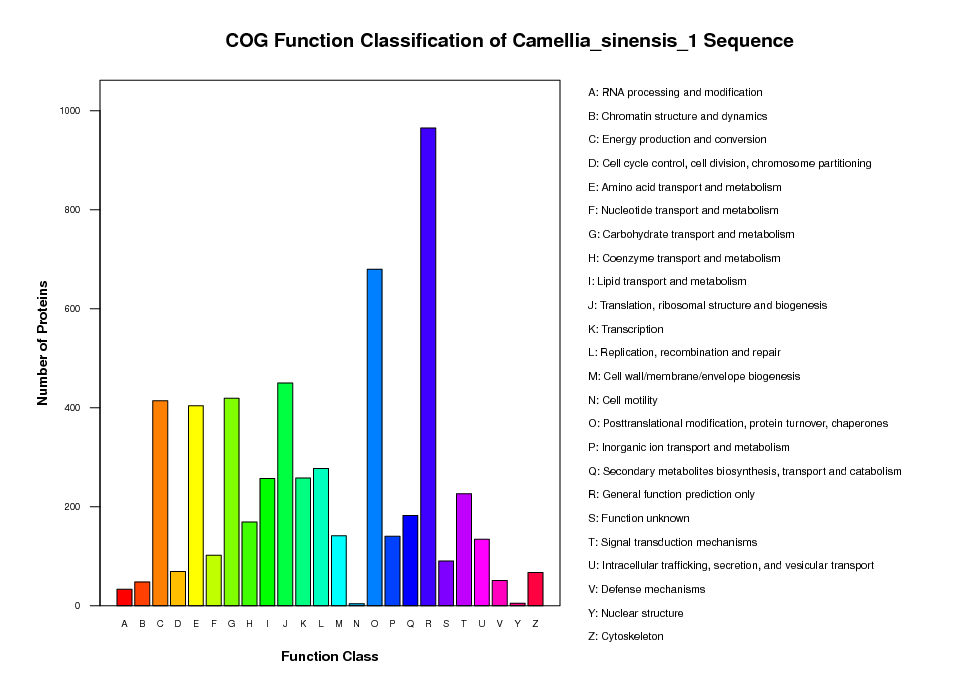


**Fig. S3.** The Gene Ontology (GO) analysis (A) and Cluster of Orthologous Groups of proteins (COG) function classification of proteins (B) in MeJA-primed fresh tea leaves with 0, 12, 24, and 48 h time duration.


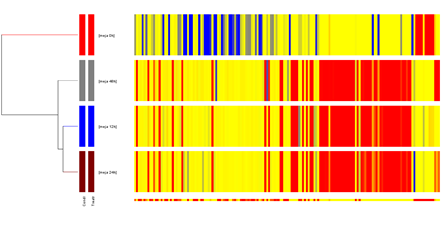

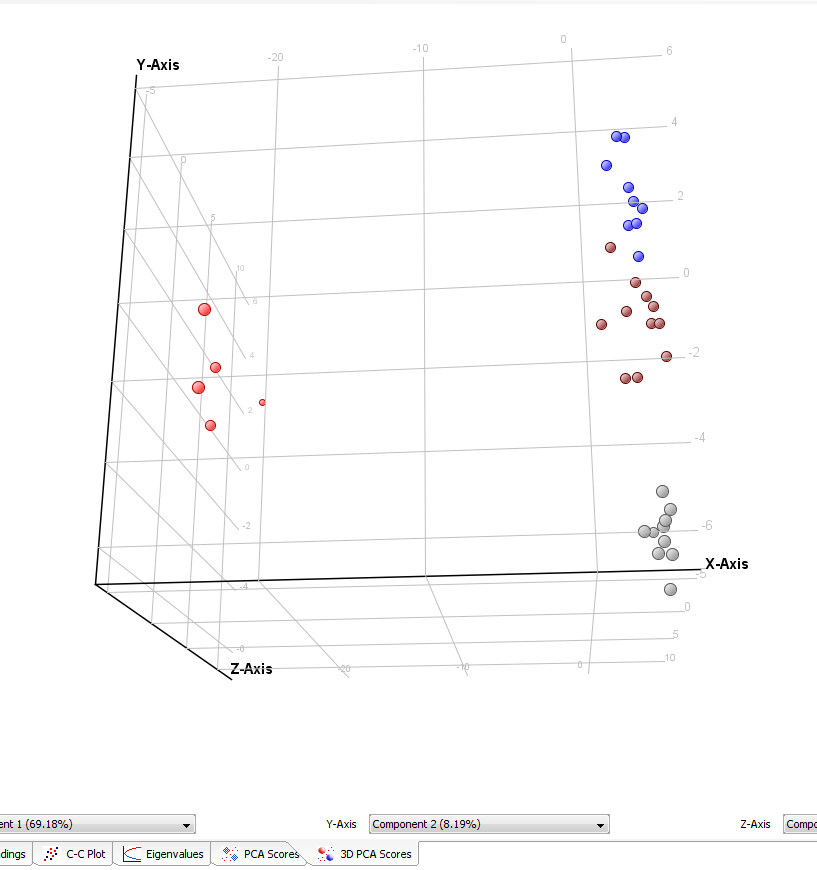


**0 h**

**12 h**

**24 h**

**48 h**

**48 h**

A

B

**24 h**

**12 h**

**0 h**

**Fig. S4.** Multivariate statistical analysis of non-volatile secondary metabolites in MeJA-primed fresh tea leaves with 0, 12, 24, 48 h and duration.

**Fig. S5.** Multivariate statistical analysis of volatile secondary metabolites in MeJA-primed fresh tea leaves with 0, 12, 24, and 48 h duration.


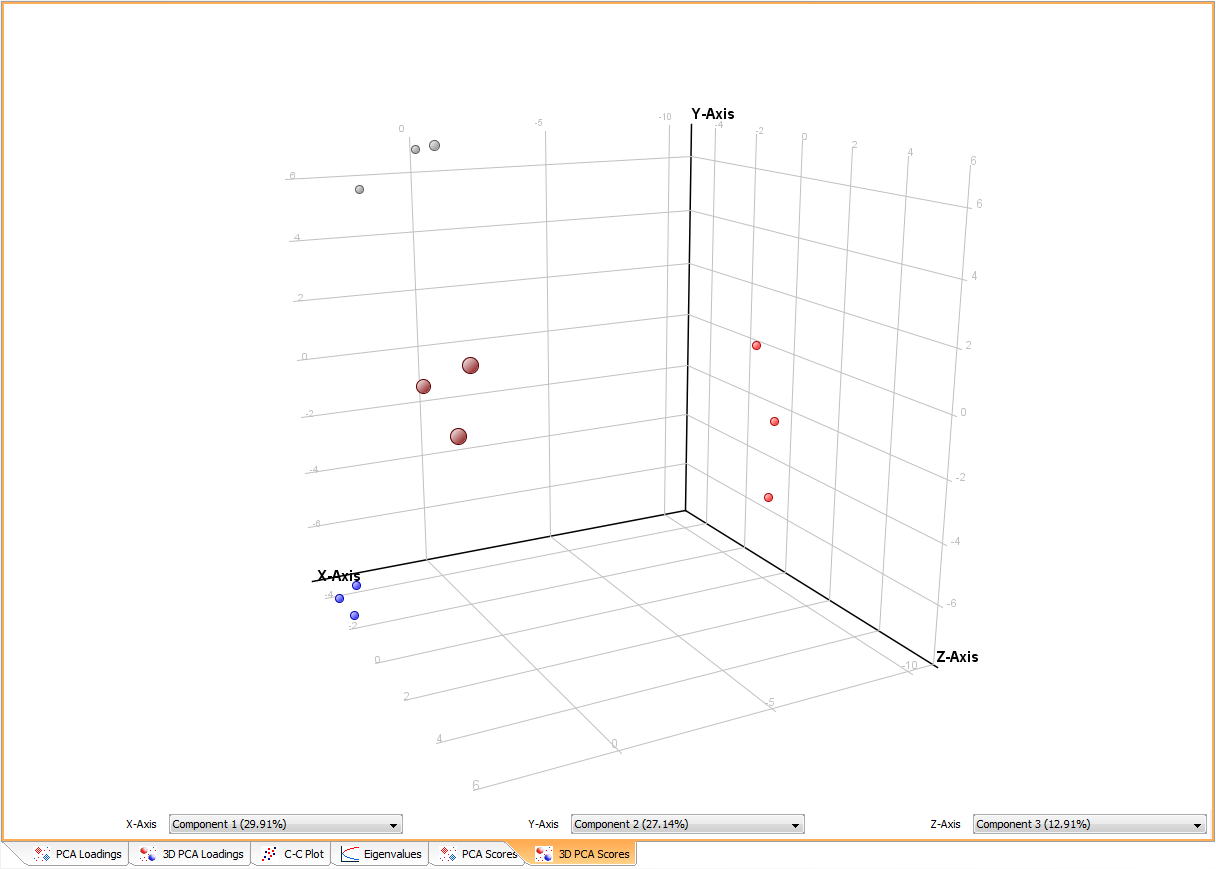

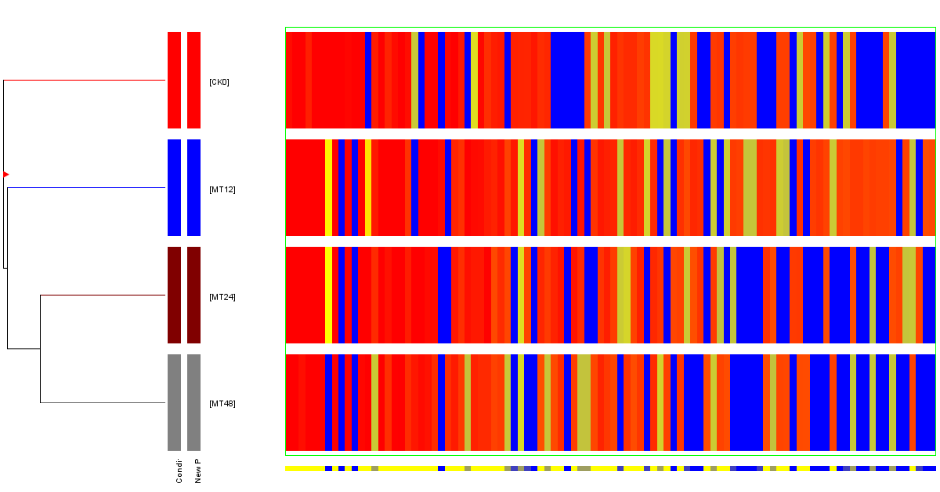


**0 h**

**12 h**

**24 h**

**48 h**

**48 h 12 h 24 h 0 h 12h 0h**

A

B

1. Isopentenyl pyrophosphate (IPP)


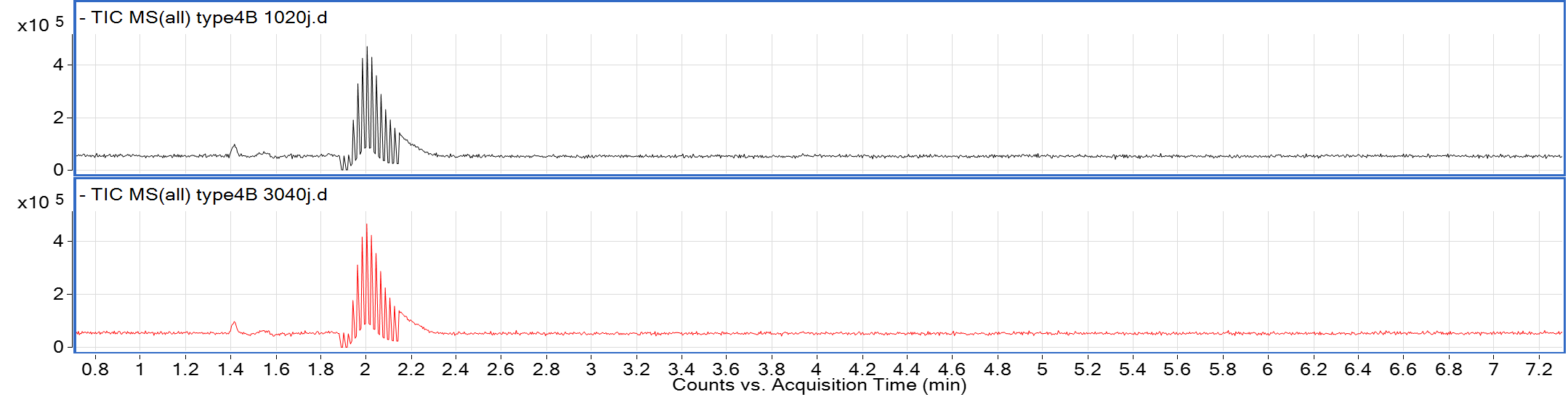


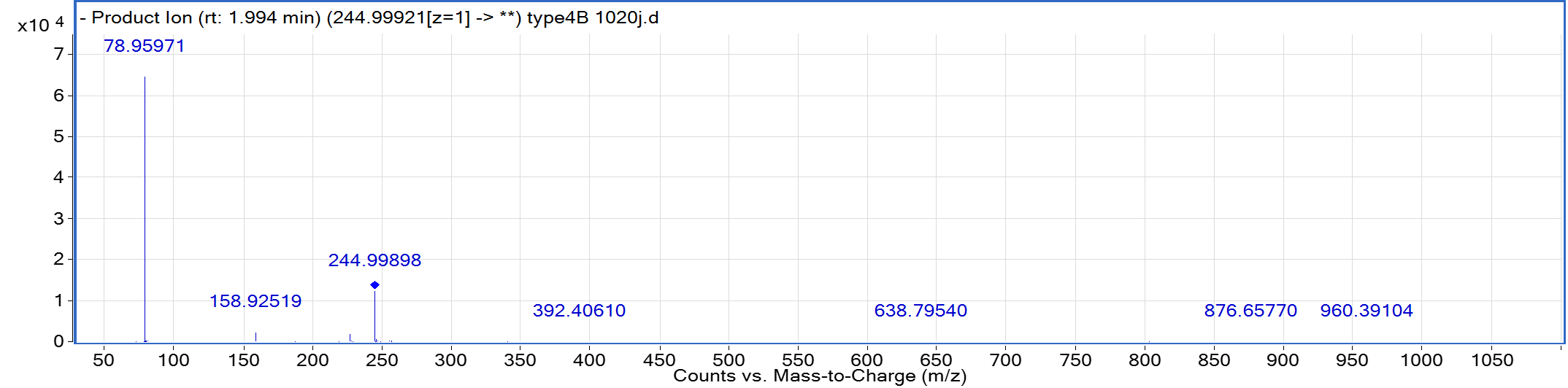


1. Dimethylally pyrophosphate (DMAPP)

1. Farnesyl diphosphate (FPP)


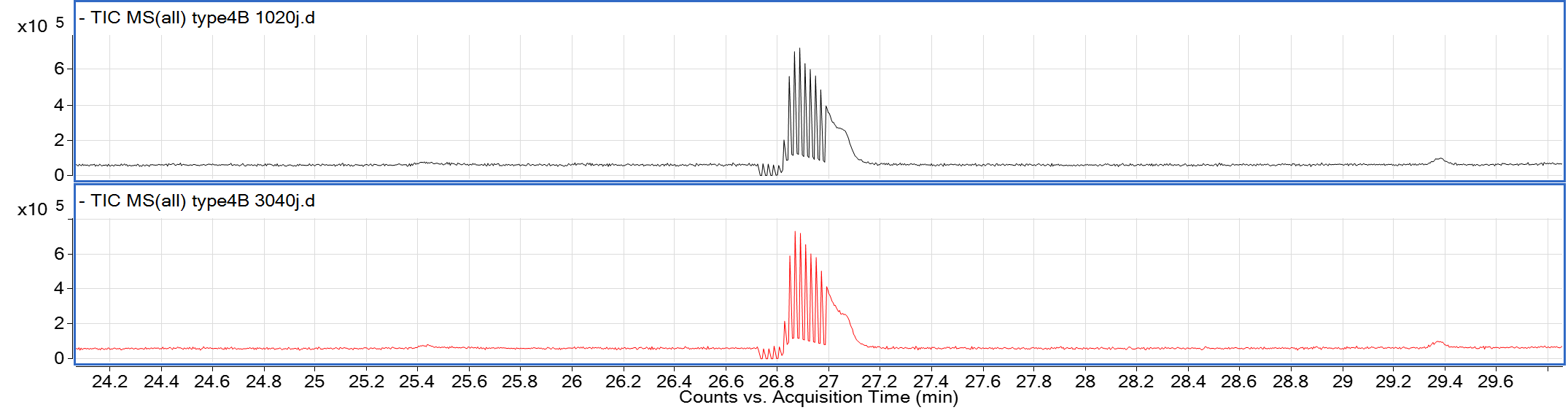


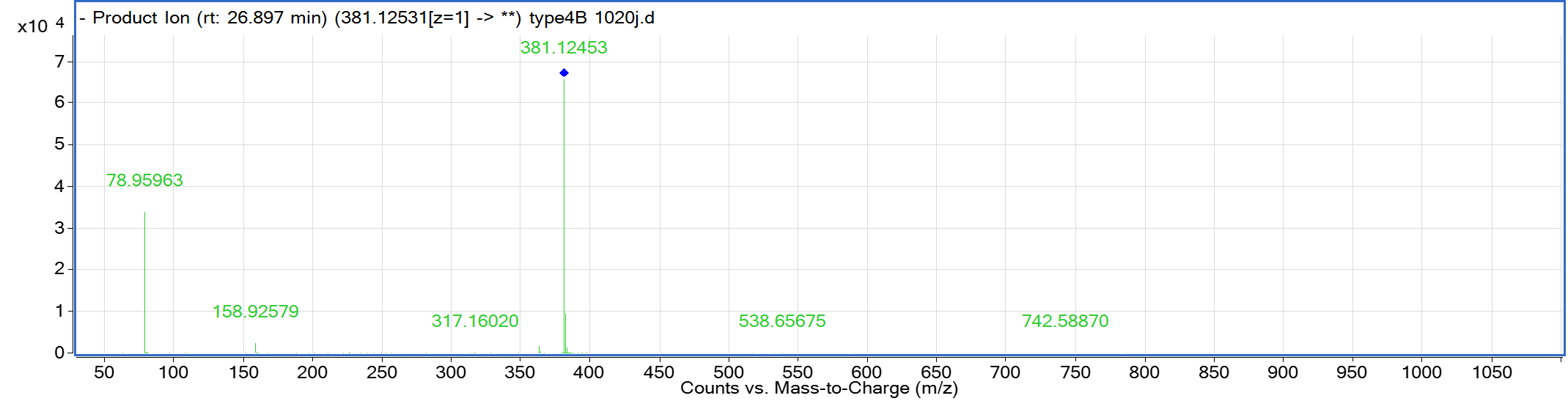


1. Geranyl diphosphate (GPP)

1. Geranylgeranyl diphosphate (GGPP)

1. *L*-Phenylalanine

1. Cinnamic acid (CA)

1. Salicylic acid (SA)

1. Ferulic acid (FA)

1. Sinapic acid

1. *p*-Coumaric acid (3 -OH cinnamic acid)

1. *trans*-3-Coumaric acid (2-OH cinnamic acid)

**Fig. S6.** MS/MS spectrometry information of 12 nonvolatile standards.
